# Myocardial Assessment with Cardiac CT: Ischemic Heart Disease and Beyond

**DOI:** 10.1007/s12410-018-9456-2

**Published:** 2018-06-02

**Authors:** Bryan C. Ramsey, Emilio Fentanes, Andrew D. Choi, Kelley R. Branch, Dustin M. Thomas

**Affiliations:** 1 Cardiology Division, Department of Medicine, San Antonio Military Medical Center, San Antonio, TX USA; 2 Cardiology Division, Department of Medicine, Tripler Army Medical Center, Honolulu, HI USA; 3 Division of Cardiology, Department of Radiology, The George Washington University School of Medicine, Washington, DC USA; 4 Cardiology Division, University of Washington, Seattle, WA USA

**Keywords:** Cardiac CT, CT perfusion, Myocardial assessment, Cardiomyopathy, Dual-energy CT

## Abstract

**Purpose of Review:**

The aim of this review is to highlight recent advancements, current trends, and the expanding role for cardiac CT (CCT) in the evaluation of ischemic heart disease, nonischemic cardiomyopathies, and some specific congenital myocardial disease states.

**Recent Findings:**

CCT is a highly versatile imaging modality for the assessment of numerous cardiovascular disease states. Coronary CT angiography (CCTA) is now a well-established first-line imaging modality for the exclusion of significant coronary artery disease (CAD); however, CCTA has modest positive predictive value and specificity for diagnosing obstructive CAD in addition to limited capability to evaluate myocardial tissue characteristics.

**Summary:**

CTP, when combined with CCTA, presents the potential for full functional and anatomic assessment with a single modality. CCT is a useful adjunct in select patients to both TTE and CMR in the evaluation of ventricular volumes and systolic function. Newer applications, such as dynamic CTP and DECT, are promising diagnostic tools offering the possibility of more quantitative assessment of ischemia. The superior spatial resolution and volumetric acquisition of CCT has an important role in the diagnosis of other nonischemic causes of cardiomyopathies.

## Introduction

Cardiovascular disease remains the worldwide leading cause of morbidity and mortality accounting for up to 31% of all deaths [1]. This trend continues to drive efforts to develop advanced detection and therapeutic modalities in hopes of stemming this pattern. Increased focus on improved diagnostic techniques has fueled a rapid expansion in advanced cardiovascular imaging techniques over the last two decades. Cardiac CT (CCT), specifically coronary CT angiography (CCTA), has been well established for the evaluation of symptomatic patients with stable or acute chest pain and concern for coronary artery disease (CAD) [2, 3]. Numerous studies have demonstrated a very high negative predictive value (~ 99%) for the exclusion of CAD. Conversely, the positive predictive value of CCTA is modest (60–80% depending on the study) in patients with a high pretest probability of obstructive CAD or those with unfavorable conditions for high-quality imaging such as rapid heart rates and significant plaque calcifications [4]. The diagnostic power of gadolinium-enhanced cardiac magnetic resonance (CMR) in the evaluation of ischemic heart disease and cardiomyopathies has been well established and is the preferred diagnostic test when the distinction between these conditions is needed in a single study. Recent studies have demonstrated similar shared characteristics in myocardial distribution and flux between iodinated contrast and gadolinium, particularly when iodinated contrast is coupled with X-ray photon attenuation profiles within the myocardium [5•]. These findings have led to expanded applications of CCT in the evaluation of ischemic heart disease and cardiomyopathies (references in comments) [6, 7, 8••].

## CCT for Chamber Size and Function Assessment

Transthoracic echocardiography (TTE) is the most widely available and commonly used technique for assessing cardiac structure and function. However, TTE assessment may be suboptimal in certain subsets of patients, namely those with poor imaging windows due to lung disease, obesity, chest wall defects, or overlying dressings in burn and post-surgical patients. CMR imaging is a powerful adjunctive test in these patients and is the current gold standard for assessment of cardiac volumes and systolic function. Compared with TTE and CMR, CCT has superior spatial resolution with decreased but comparable temporal resolution [9, 10]. Quantification of ventricular volumes and function requires acquisition of a full cardiac cycle, or R-R interval, which requires retrospective, ECG-gated scanning in most scanner platforms. While early studies reported effective radiation doses of at least 10–14 mSv utilizing retrospective acquisition and 64-slice multidetector CT (MDCT) scanner platforms, the latest generation scanner platforms have achieved doses as low as 3.8 mSv in select patients [11–13]. In head-to-head comparison studies, CCT-derived ventricular volumes and ejection fraction (EF) have excellent correlation with CMR and may be superior to both 2D and 3D echo [14•]. When viewed in cine mode on a 3D workstation, CCT can be used for the evaluation of regional wall motion changes in both the left ventricle (LV) and right ventricle (RV). To optimize acquisition and limit contrast exposure, contrast bolus injection should be tailored to the ventricle of interest. In LV-only imaging, scan triggering and injection protocols similar to those utilized for CCTA can be utilized. If biventricular assessment is needed, special attention should be paid to the contrast injection protocol to allow for uniform contrast opacification of the chamber of interest while minimizing mixing and beam-hardening artifacts common in the right heart. This typically requires a triphasic injection protocol utilizing a standard initial contrast injection (4–6 mL/s) followed by a saline/contrast mixture (possibly at a lower injection rate of 2–3 mL/s) to maximize right-heart opacification and minimizing blood/contrast swirling, and completed with a saline bolus. CCT-derived RV measurements show excellent correlation with CMR and can be especially useful in congenital heart disease patients (such as tetralogy of Fallot) and in whom implantable cardiac devices are already present [15].

## Myocardial Imaging in Ischemic Heart Disease

### Anatomy Versus Physiology in the Evaluation of CAD

Myocardial assessment in ischemic heart disease encompasses both the anatomical assessment of the cardiac dimensions and structure as well as indirectly assessing coronary artery stenosis severity and CAD chronicity. There is a complex interaction between observed coronary anatomy (i.e., luminal stenosis) and the presence of ischemia. Published data demonstrates that a luminal stenosis ≥ 50% by CCTA correlates poorly with myocardial ischemia by either single-photon emission computed tomography (SPECT) or positron emission tomography (PET) with positive predictive value (PPV) ranging from 29 to 58% [16]. Conversely, ischemia is still present in up to 12% of patients with ≥ 50% stenosis [16]. The same is true for invasive coronary angiography (ICA). Furthermore, revascularization based on ICA stenosis alone does not reduce death or nonfatal MI compared with medical therapy [17]. Physiologic assessment with invasive fractional flow reserve (iFFR) demonstrated that an intervention guided by vessel-specific ischemia for patients with indeterminate stenosis resulted in 33% less percutaneous coronary interventions and 30% improvement in composite cardiovascular outcomes [18, 19]. Given these robust data, many suggest that iFFR is the gold standard for ischemia assessment. The ongoing ISCHEMIA trial (NCT01471522) will inform the discussion regarding outcomes with revascularization based solely on ischemia. In the meantime, CCT with CCTA is positioned as the single modality capable of simultaneously evaluating coronary artery anatomy and CAD burden and assessment of physiologic myocardial blood flow.

## Multimodality Myocardial Imaging in Ischemic Heart Disease

The last decade has witnessed a shift in the diagnostic approach for ischemic heart disease away from the utilization of a single functional testing modality followed by ICA to a patient-centered multimodality approach. This approach takes into account patient parameters, preferences, and radiation dose considerations to guide therapy. As such, providers tasked with the evaluation of ischemic heart disease need a baseline understanding of the strengths and limitations of available modalities to allow for a multimodality imaging approach to these patients.

### Single-Photon Emission Computed Tomography

SPECT is a static imaging modality that leverages differential distribution and uptake of modest energy (70–120 keV) radiotracers within the myocardium based on differences in coronary blood flow and myocardial viability. SPECT imaging, compared to iFFR, has a sensitivity of 74% and specificity of 79% for the diagnosis of significant obstructive CAD [20]. Important limitations of SPECT imaging include difficulty in diagnosing high-risk CAD in the setting of balanced ischemia (i.e., global low, but homogenous blood flow), poor spatial resolution and image quality in obese patients, and effective radiation doses that average 12–15 mSv for stress-rest protocols [21, 22]. Obesity-related artifacts can be mitigated with attenuation correction or prone imaging, though these techniques can lead to artefactual perfusion defects that require the reader to synthesize data from multiple acquisitions and can increase imaging time [23, 24]. Additionally, several academic centers have implemented protocols to reduce radiation dose to include routine use of half-dose acquisitions resulting in 5–6 mSv doses [25]. The advantages of SPECT imaging are the ability to perform testing in patients that can or cannot exercise, in virtually all heart rhythms, and in known CAD and prior coronary revascularization. Additionally, there is data demonstrating the ability of SPECT to assess viability, albeit with significantly reduced sensitivity when compared to PET or CMR [26•, 27]. Finally, dynamic SPECT techniques currently being validated offer the promise of quantifying myocardial blood flow utilizing SPECT tracers [28].

### Positron Emission Tomography

PET is a versatile nuclear imaging modality that detects high-energy (512 keV) photons that result from an annihilation interaction between a positron and a valence electron. In addition to static perfusion data, the radiotracers Rb-82 and 13N-ammonia can be used to quantify absolute coronary blood flow and coronary flow reserve [29, 30]. Viability assessment can also be performed utilizing the glucose analog fluorodeoxyglucose (FDG) by leveraging the difference in metabolic properties between infarcted and hibernating tissues. When combined with anatomic CCT imaging (CAC and/or CCTA), the diagnostic performance of PET imaging for the diagnosis of CAD is greatly increased with a reported sensitivity of 90% and specificity of 95% [31]. The radiation cost of PET is modest at 2–4 mSv with the primary limitation to more widespread use of this technology limited primarily by the cost, limited scanner locations, limited available readers, and unavailability or expense of stress radiotracers.

### Cardiac Magnetic Resonance

CMR is the gold standard for the assessment of cardiac structure and function. Additionally, with emerging applications such as T1 mapping, CMR is the best validated noninvasive modality for tissue characterization. The addition of intravenous gadolinium allows for both first-pass stress imaging, utilizing gradient echo sequences, for the assessment of myocardial ischemia [32, 33]. Compared to SPECT and ICA, stress CMR assessment of ischemia was found to have a sensitivity of 89% for both and specificity of 76 and 87%, respectively [21, 34–37]. Performance of late gadolinium enhancement (LGE) sequences provides information on the presence and location of myocardial infarction, as well as robust prognostic information. Additionally, the transmural extent of LGE uptake serves as a powerful tool in the evaluation of viability. Beyond the evaluation of ischemic heart disease, mid-myocardial and/or epicardial uptake of LGE can also signal the presence of other infiltrative and inflammatory cardiomyopathies, such as sarcoidosis or idiopathic myocarditis. CMR with or without stress has its limitations. Notably, it is an expensive, time-consuming exam (often requiring 30–60 min), is poorly tolerated in patients with severe claustrophobia, and requires multiple (sometimes prolonged) breath holds, and gadolinium should not be used in patients with renal dysfunction (GFR < 30). Additionally, the presence of ferrometallic materials within the myocardium can create signal voids and limit the diagnostic utility of CMR even in those with MR conditional devices.

## CCT in the Assessment of Ischemic Heart Disease

CCT is an emerging application with the potential to deliver coronary anatomy and functional significance in a single scan. Utilizing vasodilator stress agents, CCT is able to assess differences in myocardial distribution of iodinated contrast, a technique referred to broadly as cardiac CT perfusion (CTP) [38•]. CTP protocols can differ based on the scanner platform being used, the information that is needed, and the desired patient throughput. Based on the protocol selected, the possibility exists to obtain detailed coronary anatomy (with CCTA), either first-pass (dynamic) or static stress perfusion information, stress and/or resting wall motion and EF, and CT delayed enhancement (CTDE) for the detection of myocardial infarction. Additionally, newer CT applications, such as dual-energy CT (DECT), show significant promise in the ability to further discriminate myocardial contrast uptake by leveraging the differences in attenuation profiles between tissues and contrast agents at different tube voltages. The accuracy of static CTP imaging (Table 1) compared to SPECT for predicting obstructive CAD on ICA is up to 96% sensitivity and 98% specificity, on a per vessel basis, with a PPV up to 94% and a negative predictive value (NPV) up to 98% [39, 40, 42, 43, 45–48, 63, 64]. CTP has a sensitivity and specificity of 82 and 87% compared to stress CMR, respectively, for the detection of myocardial ischemia [65]. The addition of CTDE allows for the assessment of myocardial viability with reported sensitivities of 72–77% and specificities of 88–92% when compared to LGE by CMR [66]. The following sections expand upon CTP protocol selection, post-processing considerations, and CTP techniques.

**Table 1:** Review of current CTP literature

Author (year)	No. of patients	CT scanner	Comparator	Sensitivity %	Specificity %	PPV %	NPV %
Static							
Blankstein et al. (2009) [39]	34	64-slice DSCT	SPECT	84	80	71	90
Rocha-Filho et al. (2010) [40]	35	64-slice DSCT	QCA	91	91	86	93
Feuchtner et al. (2011) [41]	30	128-slice DSCT	Stress CMR	96	88	93	94
Cury et al. (2011) [42]	26	64-detector	SPECT	94	78	89	87
Ko et al. (2012) [43]	42	320-detector	SPECT	76	84	82	79
Ko et al. (2012) [44]	40	320-detector	iFFR	74	66	56	81
George et al. (2012) [45]	50	320-detector	SPECT	50	89	55	87
Nasis (2013) [46]	20	320-detector	QCA w/ SPECT	94	98	94	98
Rochitte et al. (2014) [47]	381	320-detector	SPECT and ICA	80	74	65	86
Osawa et al. (2014) [48]	145	128-slice DSCT	ICA	85	94	79	96
Cury et al. (2015) [38•]	110	Multivendor	SPECT	90	84	36.67 reversible.fixed	99.97 reversible.fixed
Dynamic							
Kido et al. (2008) [49]	14	16-detector	SPECT	87	79	50	96
Bastarrika et al. (2010) [50]	10	128-slice DSCT	Stress CMR	86	98	94	96
Ho et al. (2010) [51]	35	128-slice DSCT	SPECT	83	78	79	82
Bamberg et al. (2011) [52]	33	128-slice DSCT	iFFR	93	87	75	97
So et al. (2012) [53]	26	64-detector	MPR vs. SPECT	95	35	83	67
Wang et al. (2012) [54]	30	128-slice DSCT	SPECT and ICA	85/90	92/81	55/58	96/96
Weininger et al. (2012) [55]	20	128-slice DSCT	Stress CMR	86	98	94	96
Rossi et al. (2013) [56]	80	128-slice DSCT	iFFR	88	90	77	95
Greif et al. (2013) [57]	65	128-slice DSCT	iFFR	95	74	48	98
Huber et al. (2013) [58]	32	256-detector	iFFR	76	100	10	91
Bamberg et al. (2014) [59]	31	128-slice DSCT	Stress CMR	78/100	75/75	51/92	91/100
Magalhaes et al. (2015) [60]	381	320-detector	SPECT and ICA	98/58	96/86	96/55	98/87
Baxa et al. (2015) [61]	54	128-slice DSCT	ICA	97	95	95	98
Wichman et al. (2016) [62]	71	128-slice DSCT	Visual assessment	100	88	43	100

Summary of data supporting CTP utilizing both static and dynamic protocols

*ICA* invasive coronary angiography, *iFFR* invasive fractional flow reserve, *CMR* cardiac magnetic resonance imaging, *SPECT* single-photon emission computed tomography, *QCA* quantitative coronary assessment/analysis, *MPR* myocardial perfusion reserve, *DSCT* dual-source CT

## CT Perfusion Protocols

**Fig. 1:**
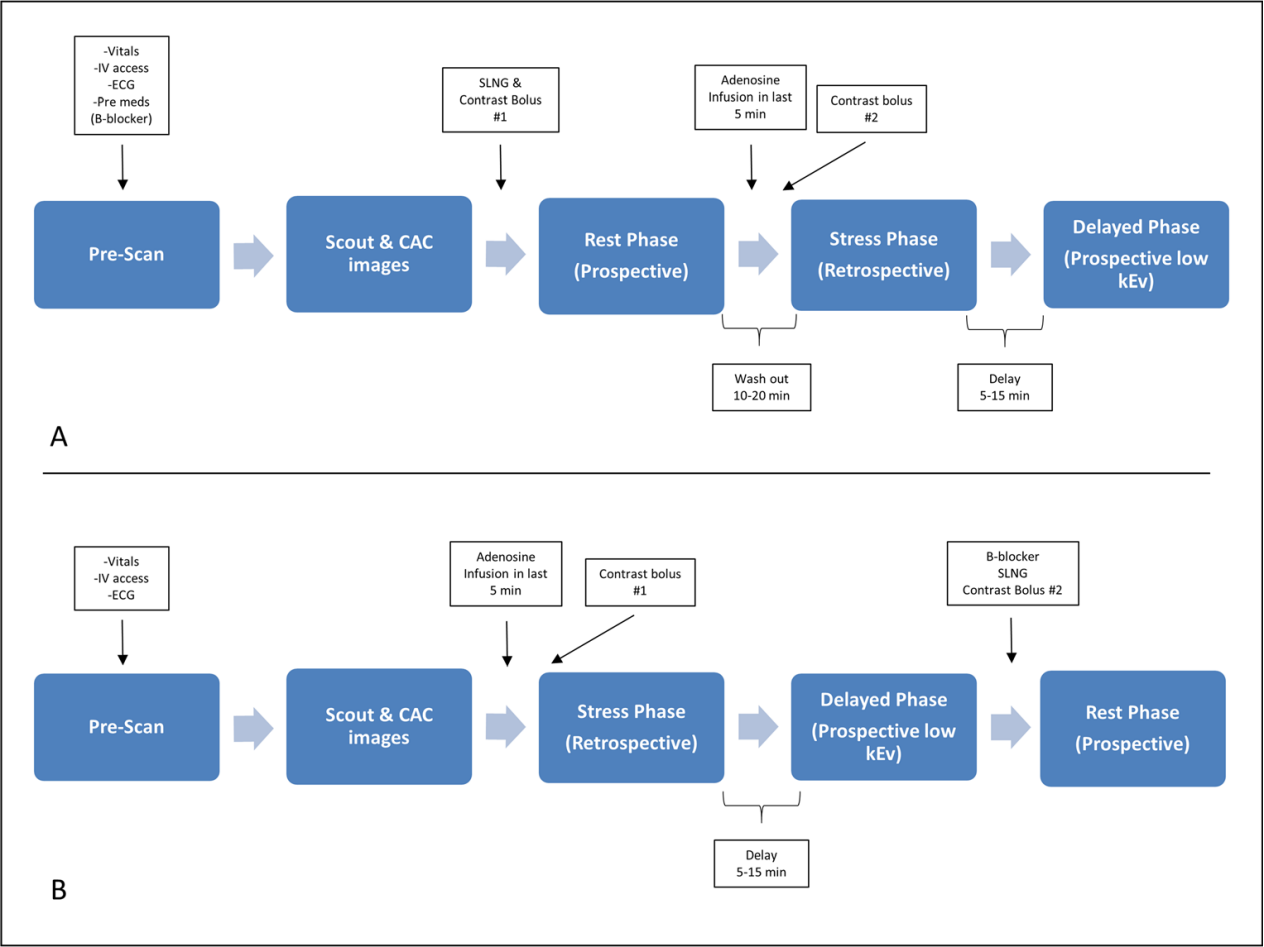
Graphical representation of two of the most common CTP protocols used. **a** Rest-stress protocol—standard patient preparation for CCTA is recommended prior to the acquisition of rest images. Vasodilator infusion can be started within the last 3–5 min of the washout phase to facilitate throughput. Finally, a 5–15-min delay is standard prior to prospective ECG-triggered acquisition for DE assessment. Total time protocol time is approximately 20–40 min. **b** Stress-rest protocol—vasodilator stress agent is given upfront followed by retrospective ECG-gated acquisition (may vary based on scanner platform). Adenosine is preferred given its short half-life, preventing carryover hyperemia and hemodynamic changes into the rest acquisition. After a 5–15-min delay, DE images can be obtained (IV nodal blocking agents can be given prior to acquisition if needed). Finally, additional nodal blockers are administered followed by nitroglycerin prior to ECG-triggered prospective rest series acquisition

**Fig. 2:**
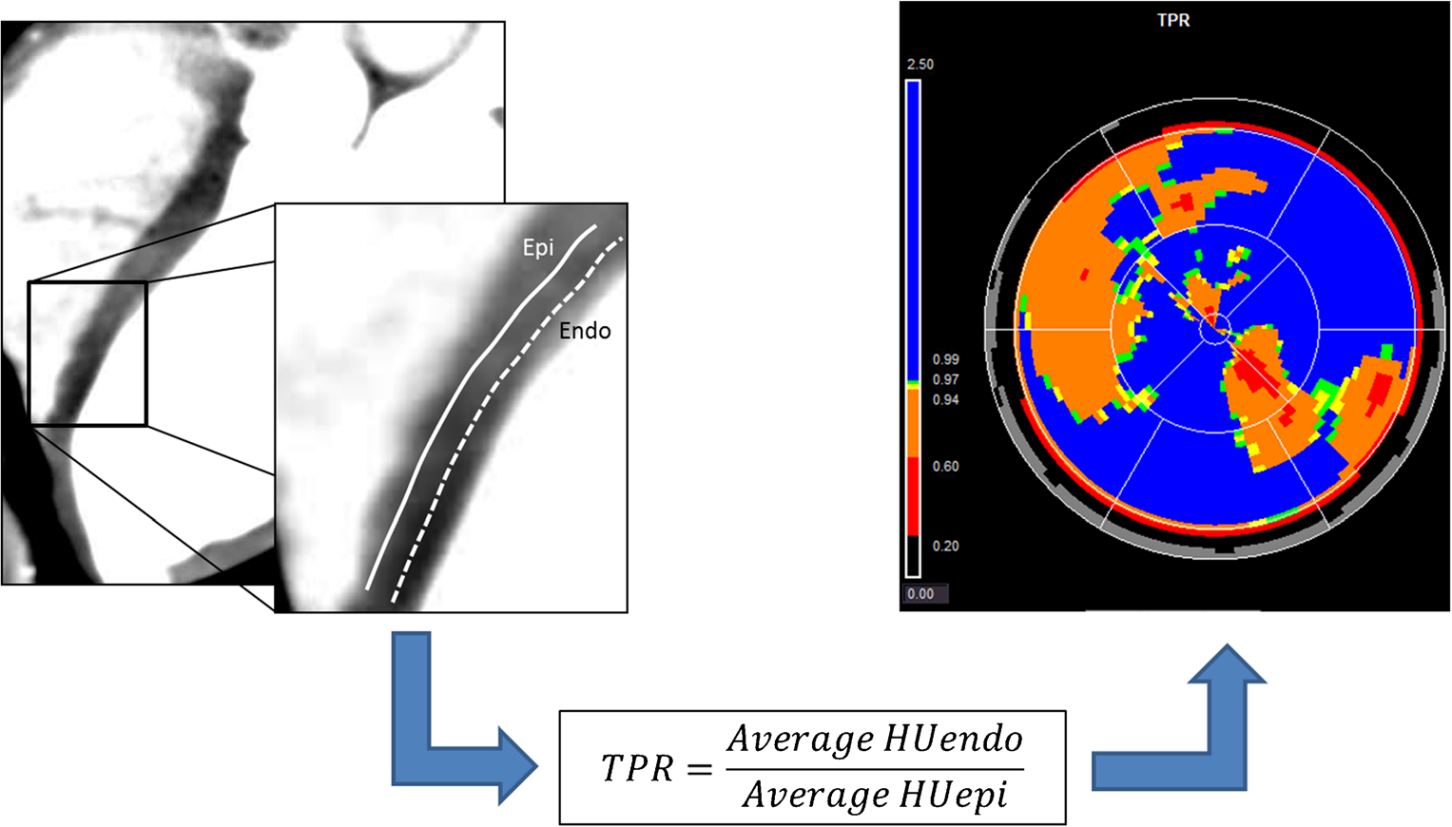
The left-sided images depict a thick-slab three-chamber average attenuation reconstruction (WW/WL 300/150) with a segment of the apical septal wall segment magnified to better demonstrate where epicardial (epi) and subendocardial (endo) regions of interest (ROI) would be drawn. TPR is calculated by obtaining the average Hounsfield unit (HU) attenuation from a ROI within the endo (HUendo) and dividing by the average HU derived from a ROI within the epi (HUepi) within the same wall segment. A ratio < 1.0 is abnormal and ratios ≤ 0.75 are highly suggestive of ischemia. The right-sided image represents available postprocessing application software available through various vendors that allow for semiautomated calculation of TPR throughout the entire myocardium. Color overlay can be added to assist with visual assessment of ischemia. In the presented image, there is evidence of ischemia in the LAD distribution. Of note, the apparent perfusion defect in the inferolateral wall segment represents a common artifact observed in CTP and not true ischemia in the left circumflex distribution

CTP relies on the kinetic properties of iodinated contrast as it is distributed and taken up into myocardial tissue. CTP imaging involves rest and stress acquisitions and can be performed in a static or dynamic method. Figure 1 depicts the most commonly used CTP protocols, which apply both to static and dynamic CTP acquisitions. Static CTP imaging refers to imaging that takes place at or near peak contrast opacification of the left heart and involves acquisition of a single dataset. Dynamic CTP imaging takes sequential datasets during the initial pass of iodinated contrast from the venous to arterial circulation. On both static and dynamic CTP imaging, regions of hypoperfusion will appear as low attenuation regions within a vascular distribution, typically worse in the subendocardial layer than the epicardial layer. In addition, software packages available within the 3D workstation may allow for generation of attenuation-based color mapping and attenuation indexing, as well as a semiquantitative assessment using a transmural perfusion ratio (TPR). TPR is simply the ratio of the average Hounsfield unit (HU) attenuation of a region of interest (ROI) within the subendocardial layer compared with the average HU attenuation within the same ROI of the epicardial layer (Fig. 2). This approach highlights the well-described phenomena of an ischemic gradient worse in the subendocardial myocardial layers and gradually improving moving closer to the epicardial coronaries. The use of TPR in static CTP significantly improves diagnostic accuracy when compared to other techniques [45, 67].

### Rest-Stress Static CTP

Rest first, followed by stress image acquisition protocol, is the most widely used in clinical practice and is best suited for low- to intermediate-risk patients without known CAD (Fig. 1a). This protocol involves an initial rest acquisition similar to simple CCTA in which an initial CAC followed by a prospective, ECG-triggered, contrast-enhanced CCTA is obtained first. Inherent in this is the fact that patients are prepped in a standard fashion with nodal blocking agents and sublingual nitroglycerin. If an indeterminate stenosis is detected, a vasodilator stress dataset is subsequently obtained. Depending on the scanner platform being used, this will either entail a retrospective, ECG-gated acquisition or, on wide-detector scanner platforms, a full R-R interval acquisition. This allows for assessment of any stress-induced wall motion changes. Finally, a delayed, noncontrast-enhanced dataset can be added approximately 10 min after the stress acquisition to evaluate for evidence of infarction. The advantage to this approach is the deferral of the stress acquisition when rest images either show nonobstructive CAD (no stenosis ≥ 50%) or a high-grade stenosis (≥ 70%). If stress imaging is pursued, a delay of 10–20 min following rest imaging should be implemented to ensure adequate contrast washout. ECG-based tube current modulation is recommended to reduce radiation dose [68]. In addition to the evaluation of stable chest pain in the outpatient setting, rest-stress CTP protocols may be ideal for the evaluation of acute chest pain in the emergency department, leveraging both the quality data and high NPV of CCTA in the ED with the ability to further evaluate indeterminate lesions and incrementally increase appropriate disposition [69]. The main limitations to rest-stress CTP protocols are the need to pretreatment with nitroglycerin and nodal blocking agents prior to rest acquisitions, which can mask ischemia, similar to data seen in SPECT imaging [70]. Additionally, residual circulating contrast from rest imaging can contaminate the stress acquisition and hinder the diagnostic performance.

### Stress-Rest Static CTP

Less commonly used when compared to rest-stress, stress-first CTP is best suited for patients with intermediate to high pretest risk known intermediate/indeterminate stenosis, or prior revascularization where the assessment of ischemia in a particular vascular territory is favored over coronary anatomy (Fig. 1b). When performing stress-first CTP, the pharmacokinetics of the vasodilatory agents being used must be taken into account. Dipyridamole, adenosine, or regadenoson can all be used and achieve hyperemia at various time periods following administration and sustain hyperemia for variable durations. Adenosine, owing to its rapid metabolism and thus rapid offset with cessation of infusion, was used in a majority of the validation studies. Regadenoson is also a viable option and is the preferred agent in SPECT and CMR due to ease of administration and a low side effect profile. The limitation of regadenoson stress-first CTP is to the persistence of heart rate elevation (30–40 min following regadenoson administration), making motion-free imaging of the coronaries challenging. Newer CT scanners can overcome the heart rate elevation associated with regadenoson with the use of motion correction software and faster gantry rotation speeds allowing for stress-only CTP and high-resolution coronary anatomy in a single, stress acquisition, mitigating the need for rest acquisition and thus conserving radiation dose.

### Dynamic (First-Pass) CTP

Static imaging techniques, with or without stress acquisitions, are limited to single snapshots in time and do not provide comprehensive blood flow analysis. Historically, limitations in scanner technology made static CTP the only viable method. However, the latest generation 256- and 320-row detector platforms allow for imaging of the entire cardiac volume with a stationary table and a single gantry rotation. Additionally, second-generation dual-source CT (DSCT) can cover this same volume utilizing a table shuttle method. The third-generation DSCT has increased *z*-axis coverage up to 105 mm and, thus, can image the cardiac volume without the need for table shuttling [50, 51, 55, 71]. This technology allows for the performance of first-pass perfusion owing to the ability of these newer generation scanners to acquire full cardiac datasets in short succession, termed dynamic CTP. Dynamic CTP allows for comparison of time-attenuation profiles within myocardial segments, which facilitates direct quantification of myocardial blood flow (MBF) [72]. MBF calculation by dynamic CTP involves mathematic modeling derived from the deconvolution methods used in CMR [52, 73]. In semiquantitative analysis, the time-attenuation curve for a myocardial ROI is derived and a time-to-peak attenuation, attenuation upslope, and area under the curve are calculated. This is the most commonly used semiquantitative method as only the upslope time to peak attenuation is sampled, thus lowering effective radiation dose. Dynamic CTP validation studies, utilizing 320-row MDCT and second-generation DSCT, have shown varying, but mostly positive results in detection of hemodynamically significant CAD when compared against ICA, CMR, and SPECT. Dynamic CTP (Table 1) has demonstrated sensitivities ranging from 58 to 100%, specificities from 74 to 100%, NPV 82–100%, and PPV 43–100% [51, 56, 57, 60]. The biggest limitation of dynamic CTP is the relatively high radiation dose required (8.2 to 18.8 mSv in validation studies) [62, 73]. Dynamic CTP represents an emerging CCT application and further research is needed before more widespread implementation is pursued.

### Dual-Energy Computed Tomography

**Fig. 3:**
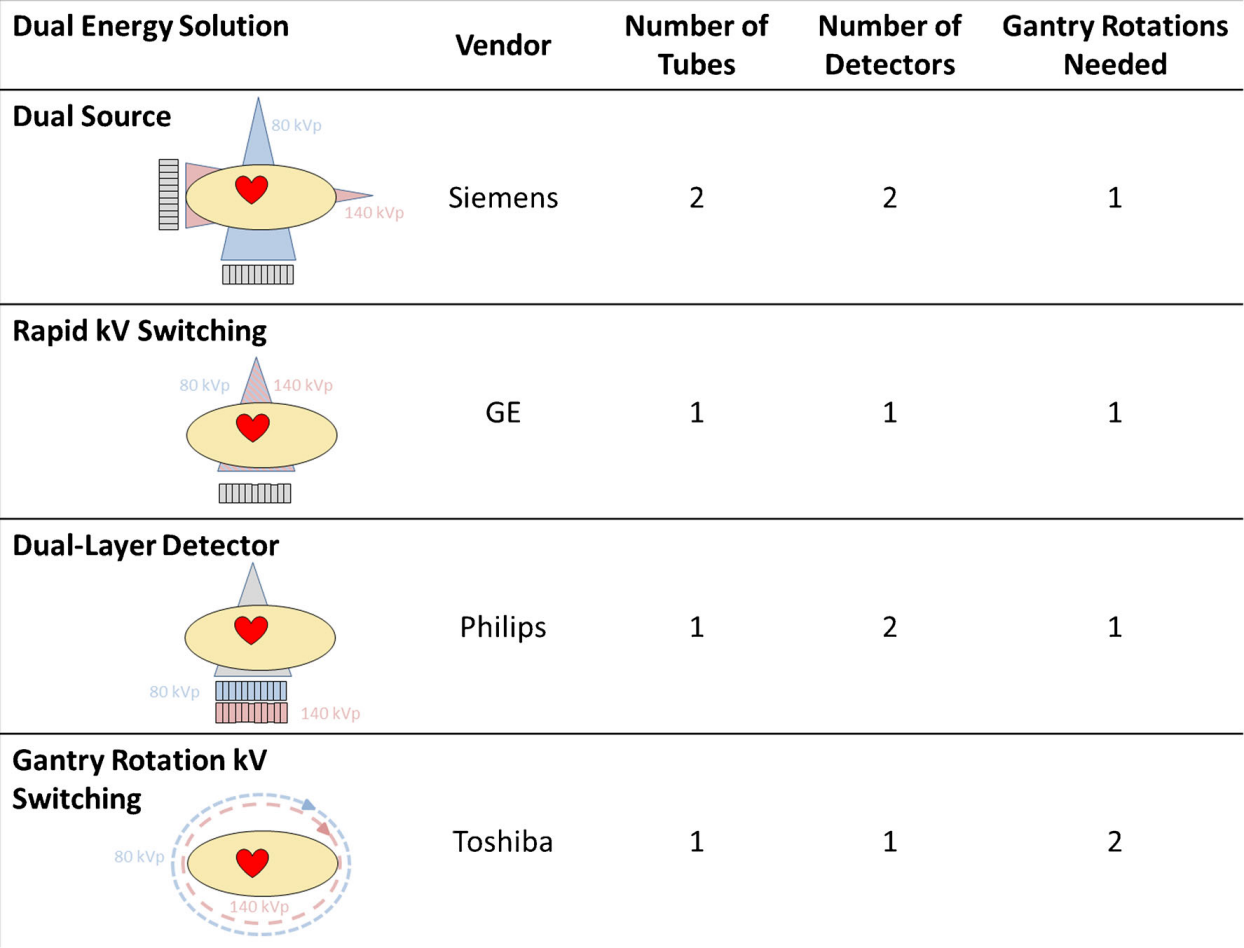
Representation of currently available vendor-specific dual-energy CT (DECT) solutions available to date

DECT was first introduced in 2008 and has undergone several advancements and innovations in the last decade that have significantly increased its diagnostic utility [74, 75]. DECT is based on the principles of the photoelectric effect and the energy-related attenuation difference of tissues observed with exposing the same sample volume to both a low (typically 80 kV) and high (140 kV) tube voltage. Utilizing monochromatic reconstructions at these differing energy levels, subtle differences in tissue contrast uptake can be more readily detected. Specific to CTP, DECT facilitates creating of an iodine map that serves as a surrogate for blood flow [76]. This is accomplished by utilizing one of four vendor-specific technologies (Fig. 3): two X-ray sources offset by 90° operating at different energy levels, rapid switching utilizing a single source where the X-ray tube cycles rapidly between low and high tube voltage during a single gantry rotation, a dual layer detector model where a single X-ray source provides a spectrum of energy levels in the presence of a double-layered detector configuration that registers only high- and low-energy photons, and gantry rotation kilovolt switching where a single X-ray source scans a full gantry rotation at high- and a full gantry rotation at low-energy settings of the same tissue volume (thus each volume is scanned twice) [77]. With these specialized acquisitions, a virtual monochromatic image (VMI) is generated that is less susceptible to beam hardening and other artifacts while maximizing the superior contrast seen with iodinated agents and soft tissue at low kilovolt settings [78–80]. DECT can readily delineate the iodinated contrast in the blood pool within the ventricle and within the vessels and absorbed by the myocardium and can then be used to make color-coded maps, similar to SPECT images, that detail myocardial perfusion [76, 81]. Compared with SPECT and single-energy CTP, DECT protocols (Table 2) are observed to have a sensitivity of 82–94%, specificity of 71–94%, PPV of 53–91%, and NPV of 81–97% [84, 85]. Historically, one of the main limitations to DECT was the high required radiation dose and high contrast volume [86]. However, subsequent advancements have shown that the use of ultralow-energy levels (40–50 kV) enhances iodine contrast differences and improves the accuracy of delayed enhancement imaging, particularly for the detection of scar [87]. Several studies of DECT have achieved radiation doses of 0.5 to 4.4 mSv, significantly reduced when compared to early DECT or SPECT [88, 89]. Additionally, no reduction in image quality was observed despite reductions in contrast volume approaching 50% [90, 91]. Currently, DECT for myocardial perfusion is not routinely utilized in clinical practice as further study is ongoing to determine the optimal energy settings and to further investigate the various vendor-specific DECT solutions more thoroughly for cardiac imaging [92–94].

**Table 2:** Review of current literature supporting dual-energy CTP

Author (year)	No. of patients	CT scanner	Comparator	Sensitivity %	Specificity %	PPV %	NPV %
Ruzsics et al. (2009) [74]	36	64-slice DSCT	SPECT	92	93	83	97
Wang et al. (2011) [82]	31	64-slice DSCT	Stress CMR	89	78	74	91
Ko et al. (2011) [83]	50	64-slice DSCT	Stress CMR	89	78	74	91
Ko et al. (2012) [43]	45	64-slice DSCT	ICA	89	74	80	85
Kim et al. (2014) [84]	50	128-slice DSCT	Stress CMR	94	71	60	96

Summary of data supporting CTP utilizing both static and dynamic protocols

*ICA* invasive coronary angiography, *CMR* cardiac magnetic resonance imaging, *DSCT* dual-source CT

### CTP Post-processing at the 3D Workstation

**Fig. 4:**
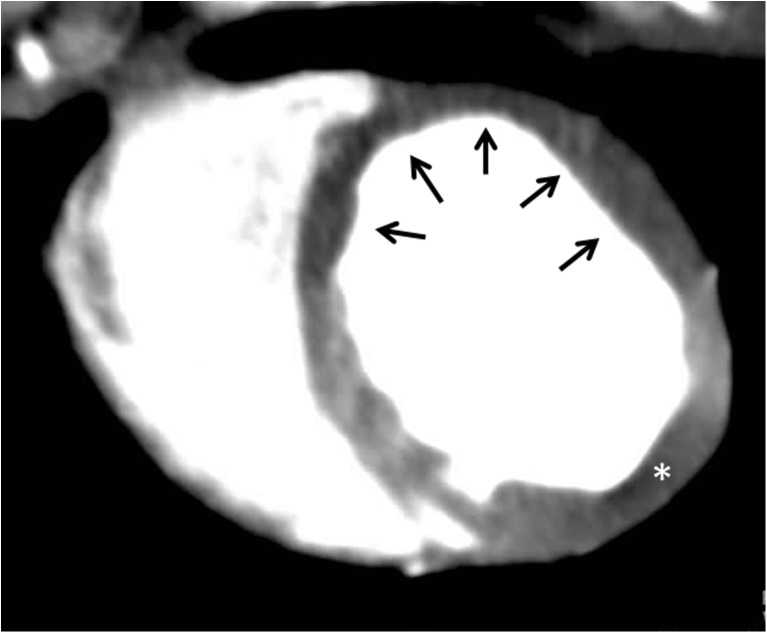
Thick-slab average HU short-axis projection demonstrating a perfusion defect in the LAD territory (black arrows). In the visual assessment of ischemia with CTP imaging, windowing at the 3D workstation is vital to maximize visual discrimination between ischemic myocardium (HU attenuation between 30 and 70) and normal myocardium (HU attenuation ~ 100). As is commonly observed, a hypoattenuation artifact is present in the inferolateral wall segment secondary to beam hardening from the descending thoracic aorta (*) mimicking a perfusion defect in this territory

Post-processing of CTP datasets relies on the visual assessment of the ischemic myocardial segments in comparison to normally perfused myocardium (Fig. 4). Multiplaner reformatted images allow for evaluation in the classic 17 segment model view. Image display settings should be adjusted to thick MPR slabs (3–8 mm) and minimum intensity projection (MinIP) or average HU attenuation projection as opposed to maximum intensity projection (MIP). This allows for more ready identification of ischemic segments. Finally, appropriate window width and level settings (200–300 and 100–150, respectively) should be utilized [39, 95]. These settings optimize the displayed grayscale centering around the normal HU attenuation of the myocardium (average HU of 90–100) and the narrow width accentuates ischemic or infarcted myocardium ranging from subzero HU to 30 HU [96, 97]. TPR (Fig. 2), as discussed above, is a semiquantitative assessment of perfusion that measures the ratio of the average HU of the subendocardial to subepicardial tissue where a normal TPR has been defined as above 1 and a ratio of 0.75 or less suggests ischemia [42]. The combination of DE-CCT with TPR compared to SPECT demonstrates a sensitivity of 86%, specificity of 92%, positive predictive value of 92%, and negative predictive value of 85% for diagnosing clinically significant perfusion defects.

#### Limitations of CTP

Radiation dose, as mentioned above, continues to be a limitation to widespread implementation of CTP protocols. Newer generation scanners and the possibility of single acquisition CCTA and stress CTP hold promise for lowering radiation dose to levels more comparable to SPECT. Imaging artifacts, specifically beam hardening from the descending thoracic aorta, can affect interpretation of the inferolateral wall segments by mimicking a perfusion defect in that territory. Utilization of beta-blockers and nitrates, as is often required for acquisition of CCTA data, reduces the sensitivity of CTP scans by masking smaller, typically single-vessel, perfusion defects as shown in the SPECT literature [98, 99]. Finally, as summarized in Fig. 1a, a 10–20-min washout period is paramount when utilizing rest-stress acquisition protocols. Iodinated contrast is slow to wash into (and subsequently out of) ischemic territories. The presence of residual contrast in the myocardium at the time of the second contrast bolus injection narrows the attenuation profile differences between normal and ischemic myocardium, thus reducing the sensitivity for detection of ischemic defects.

## Infarct Assessment Utilizing CTDE

Over the last two decades, advancement in CMR with LGE has revolutionized the assessment of myocardial fibrosis secondary to infarction, infiltration, or inflammation. The ability of CMR to assess these various tissue states is based on the pathologic effects on the tissues resulting in changes in tissue density and differential uptake of gadolinium. Iodinated contrast has similar kinetics and distribution to gadolinium allowing for the potential of DECT to detect infarction similar to CMR [100]. As mentioned above, CTDE involves the acquisition of a delayed, noncontrast-enhanced dataset obtained approximately 10 min after the last contrast-enhanced dataset. Similar to gadolinium imaging characteristics with CMR, infarcted tissues will have a delayed washout for iodinated contrast material and appear hyperenhancing [5•, 101]. Small studies have confirmed a correlation of 81–85% in the detection of infarction compared to CMR [102, 103]. The prognostic importance of DE findings on CT was assessed in a small study of 102 patients who showed a 19% rate of MACE at 2 years. Based on these results, CTDE was identified as an independent predictor of major adverse cardiovascular events (MACE) [104]. Utilization of ultralow kiloelectron volt settings can reduce artifact and accentuate smaller areas of residual contrast uptake within the myocardium, though more studies are needed [87].

## CCT in the Assessment of Nonischemic and Inheritable Cardiomyopathies

**Fig. 5:**
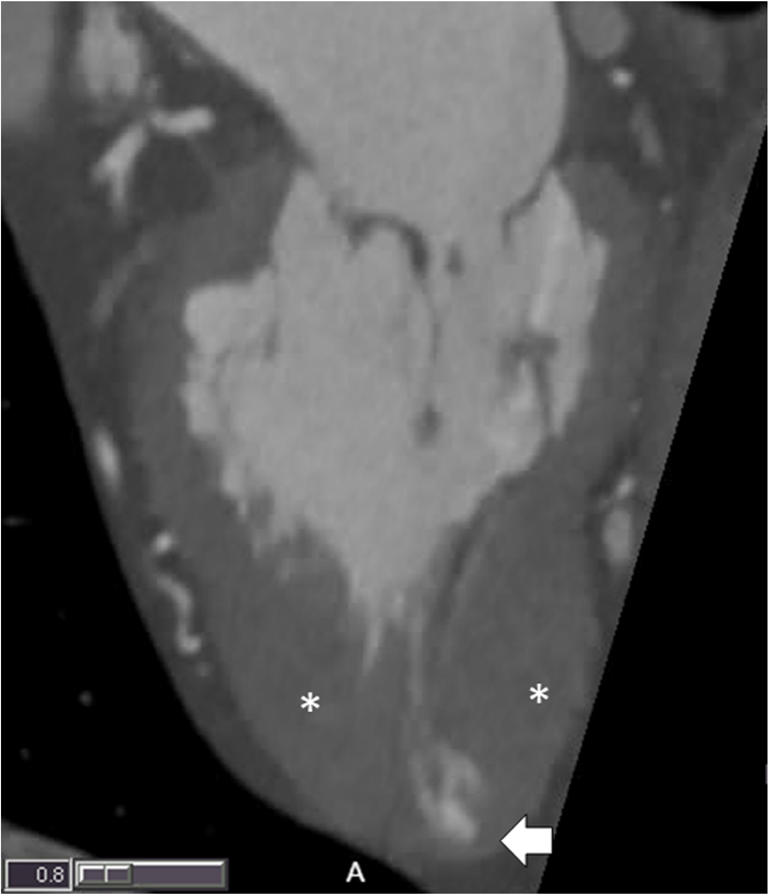
Thin-slab two-chamber projection demonstrating isolated LV apical hypertrophy (*) in a patient with the apical variant of hypertrophic cardiomyopathy. The white arrow denotes a small apical aneurysm/pouch, which is commonly observed in this variant of HCM and easily appreciated on CCT

CCT can serve as an important adjunctive modality to TTE in patients with known or suspected cardiomyopathies, primarily in patients with claustrophobia, implantable cardiac devices, and poor TTE windows. In the setting of newly diagnosed heart failure with a reduced ejection fraction, CCTA is well validated to exclude significant CAD in patients with low to intermediate pretest risk of CAD. In patients with reduced EF less than 35%, CCTA for the evaluation of CAD has a reported sensitivity of 98% and specificity of 97% [105]. While a prospective, ECG-triggered protocol is routinely used to minimize patient radiation dose, full cardiac cycle imaging allows for the assessment of wall motion and facilitates ventricular volumetric and EF assessment that correlate strongly with CMR [15, 106]. Several techniques including ECG-based tube current modulation, low and ultralow kilovolt imaging, and iterative reconstruction have been used to reduce radiation dose in retrospective acquisition of images [107]. When compared to TTE, SPECT, and CMR-based assessments, the CT-derived measurements correlate well with an observed slight overestimation of LVEF. Specific to cardiomyopathies involving the RV, scan protocol changes to the contrast bolus injection may be necessary in order to optimize RV opacification while minimizing blood-contrast mixing and beam-hardening artifacts. A triphasic contrast injection protocol involving an initial 100% contrast bolus at a rate between 4 and 6 mL/s followed by a saline/contrast mix at a lower rate (~ 2 mL/s) and terminating with a saline bolus has been shown to provide optimal right-sided chamber opacification [108]. Table 3 highlights CCT findings that can help to make a diagnosis. As outlined above, appropriate protocol selection is vital in cardiomyopathies where regional wall motion, ventricular volumes, or valve motion (SAM) is needed. As an example, Fig. 5 highlights the strengths of CCT in a patient with apical-variant hypertrophic cardiomyopathy. CCT allows for precise assessment of wall thickness and possible DE if appropriately protocoled. Additionally, the apical aneurysm/pouch commonly encountered in apical-variant HCM is easily visualized, and though not present here, thrombus formation would be easily diagnosed.

**Table 3:** Common findings by CCT in cardiomyopathies

Cardiomyopathy	CCT findings
Dilated nonischemic cardiomyopathy (NICM)	• Global systolic dysfunction • Dilated ventricle • Apical tenting of MV leaflets • Hypertrabeculation not meeting LVNC criteria • Absence of significant CAD
Hypertrophic cardiomyopathy (HCM)	• Asymmetric hypertrophy of basal interventricular septum or apex • Wall segment > 15 mm at end-diastole (> 25 mm with HTN) • SAM of the MV on cine imaging • Patchy or diffuse midmyocardial DCE
Myocarditis/myopericarditis	• Global or regional HK • ± Pericardial effusion • Midmyocardial or epicardial DCE
Sarcoidosis	• Patchy uptake of DCE • Global or regional WMA in noncoronary distribution • Focal wall thickening (acute) or wall thinning (chronic)
Amyloidosis	• Diffusely increased myocardial wall thickening • Biatrial enlargement • Diffuse subendocardial (but can have transmural) DCE
LV noncompaction	• Increased ratio of noncompacted to compacted myocardium > 2.2 in end-diastole • Involvement of > 2 segments apical to papillary muscles • NC mass of LV > 20–25% total LV mass • NC mass > 15 g/m^2^ • LV crypt thrombus
Arrhythmogenic RV cardiomyopathy (ARVC)	• Excessive mural fat content, particularly within the RV • Regional RV WMA • RV aneurysm • RV dilation (EDV > 110 mL/m^2^ males/> 100 mL/m^2^ females) • RV systolic dysfunction (RVEF < 40%)
Stress-induced cardiomyopathy (Takotsubo)	• Hyperdynamic basal wall segments • Akinetic/dyskinetic apical segments • Absence of DCE (i.e., no evidence of infarct) • SAM

List of the most commonly encountered cardiomyopathies and their correlating findings on cardiac computed tomography (CCT)

*MV* mitral valve, *LVNC* left ventricular noncompaction, *CAD* coronary artery disease, *HTN* hypertension, *SAM* systolic anterior motion, *DCE* delayed contrast enhancement, *WMA* wall motion abnormality, *NC* noncompacted, *LV* left ventricle, *RV* right ventricle, *EDV* end-diastolic volume, *RVEF* right ventricular ejection fraction

## Myocardial Assessment with Hybrid Cardiac Imaging (PET/CT)

PET when combined with CT has emerged as a powerful diagnostic modality, both in ischemic heart disease as well as various inflammatory and infiltrative disease processes. PET imaging is commonly undertaken to assess the metabolic activity of tissue utilizing the glucose analog ^18^F-fluorodeoxyglucose (FDG). FDG PET imaging, taking advantage of differences in glucose metabolism between normal myocytes and diseased myocytes, has the ability to detect hibernating myocardium in viability testing and myocyte inflammation as seen in acute cardiac sarcoidosis [109]. Imaging these very different disease states requires significant preimaging patient preparation involving standardized protocols meant to manipulate the glucose substrate environment available to myocytes [110].

## Future Applications

While the utility of DE images has been discussed as it relates to infarct detection in ischemic heart disease, iodine mapping with single- or dual-energy CT can also be employed in the assessment of other cardiomyopathies where epicardial and midmyocardial scar patterns are currently observed on CMR exclusively. CCT-based estimation of extracellular volume (ECV) by CCT may become a useful diagnostic and prognostic marker of myocardial remodeling similar to that observed with T1 mapping by CMR [111–113]. Strain or deformation imaging, a well-validated TTE for the early detection of chemotherapy-induced cardiotoxicity, can also be calculated on CCT using the velocity gradients between two points in the myocardium with comparable accuracy to that of TTE [114].

## Conclusion

CCT in the form of CTP, particularly when combined with CCTA, is a powerful tool in the assessment of ischemic heart disease and, with newer generation scanner platforms, presents the potential for full functional and anatomic assessment with a single contrast injection and low radiation dose dataset acquisition. CCT is a useful adjunct to both TTE and CMR in the evaluation of ventricular volumes and systolic function, particularly in patients with implantable cardiac devices or severe claustrophobia. Newer applications of CCT, namely dynamic CTP and DECT, are promising diagnostic tools offering the possibility of more quantitative assessment of ischemia than offered by static perfusion imaging. Finally, given its superior spatial resolution and volumetric acquisition, CCT has an important role in the diagnosis of other nonischemic causes of cardiomyopathies most notably LVNC, ARVC, and HCM.

## References

[1] Roth GA, Huffman MD, Moran AE, Feigin V, Mensah GA, Naghavi M, Murray CJL (2015). Global and regional patterns in cardiovascular mortality from 1990 to 2013. Circulation.

[2] Taylor AJ, Cerqueira M, Hodgson JM, Mark D, Min J, O'Gara P, Rubin GD, Kramer CM, Berman D, Brown A, Chaudhry FA, Cury RC, Desai MY, Einstein AJ, Gomes AS, Harrington R, Hoffmann U, Khare R, Lesser J, McGann C, Rosenberg A, Schwartz R, Shelton M, Smetana GW, Smith SC Jr, American College of Cardiology Foundation Appropriate Use Criteria Task Force, Society of Cardiovascular Computed Tomography, American College of Radiology, American Heart Association, American Society of Echocardiography, American Society of Nuclear Cardiology, North American Society for Cardiovascular Imaging, Society for Cardiovascular Angiography and Interventions, Society for Cardiovascular Magnetic Resonance (2010). ACCF/SCCT/ACR/AHA/ASE/ASNC/NASCI/SCAI/SCMR 2010 appropriate use criteria for cardiac computed tomography. A report of the American College of Cardiology Foundation Appropriate Use Criteria Task Force, the Society of Cardiovascular Computed Tomography, the American College of Radiology, the American Heart Association, the American Society of Echocardiography, the American Society of Nuclear Cardiology, the North American Society for Cardiovascular Imaging, the Society for Cardiovascular Angiography and Interventions, and the Society for Cardiovascular Magnetic Resonance. J Am Coll Cardiol.

[3] Hendel RC, Patel MR, Kramer CM, Poon M, Hendel RC, Carr JC, Gerstad NA, Gillam LD, Hodgson JM, Kim RJ, Kramer CM, Lesser JR, Martin ET, Messer JV, Redberg RF, Rubin GD, Rumsfeld JS, Taylor AJ, Weigold WG, Woodard PK, Brindis RG, Hendel RC, Douglas PS, Peterson ED, Wolk MJ, Allen JM, Patel MR, American College of Cardiology Foundation Quality Strategic Directions Committee Appropriateness Criteria Working Group, American College of Radiology, Society of Cardiovascular Computed Tomography, Society for Cardiovascular Magnetic Resonance, American Society of Nuclear Cardiology, North American Society for Cardiac Imaging, Society for Cardiovascular Angiography and Interventions, Society of Interventional Radiology (2006). ACCF/ACR/SCCT/SCMR/ASNC/NASCI/SCAI/SIR 2006 appropriateness criteria for cardiac computed tomography and cardiac magnetic resonance imaging: a report of the American College of Cardiology Foundation Quality Strategic Directions Committee Appropriateness Criteria Working Group, American College of Radiology, Society of Cardiovascular Computed Tomography, Society for Cardiovascular Magnetic Resonance, American Society of Nuclear Cardiology, North American Society for Cardiac Imaging, Society for Cardiovascular Angiography and Interventions, and Society of Interventional Radiology. J Am Coll Cardiol.

[4] Meijboom WB, Meijs MF, Schuijf JD, Cramer MJ, Mollet NR, van Mieghem CA (2008). Diagnostic accuracy of 64-slice computed tomography coronary angiography: a prospective, multicenter, multivendor study. J Am Coll Cardiol.

[5] • Gerber BL, Belge B, Legros GJ, Lim P, Poncelet A, Pasquet A, Gisellu G, Coche E, Vanoverschelde JL Characterization of acute and chronic myocardial infarcts by multidetector computed tomography: comparison with contrast-enhanced magnetic resonance. Circulation. 2006;113(6):823–33. doi:10.1161/circulationaha.104.529511. **A sentinel paper in establishing CCT imaging parameters for assessment of infarction**. 10.1161/CIRCULATIONAHA.104.529511 16461822

[6] Budoff MJ, Nakazato R, Mancini GB, Gransar H, Leipsic J, Berman DS (2016). CT angiography for the prediction of hemodynamic significance in intermediate and severe lesions: head-to-head comparison with quantitative coronary angiography using fractional flow reserve as the reference standard. JACC Cardiovasc Imaging.

[7] Budoff MJ, Li D, Kazerooni EA, Thomas GS, Mieres JH, Shaw LJ (2017). Diagnostic accuracy of noninvasive 64-row computed tomographic coronary angiography (CCTA) compared with myocardial perfusion imaging (MPI): the PICTURE study, a prospective multicenter trial. Acad Radiol.

[8] Pelgrim GJ, Dorrius M, Xie X, den Dekker MA, Schoepf UJ, Henzler T (2015). The dream of a one-stop-shop: meta-analysis on myocardial perfusion CT. Eur J Radiol.

[9] Schuleri KH, George RT, Lardo AC (2009). Applications of cardiac multidetector CT beyond coronary angiography. Nat Rev Cardiol.

[10] Budoff MJ, Dowe D, Jollis JG, Gitter M, Sutherland J, Halamert E, Scherer M, Bellinger R, Martin A, Benton R, Delago A, Min JK (2008). Diagnostic performance of 64-multidetector row coronary computed tomographic angiography for evaluation of coronary artery stenosis in individuals without known coronary artery disease: results from the prospective multicenter ACCURACY (Assessment by Coronary Computed Tomographic Angiography of Individuals Undergoing Invasive Coronary Angiography) trial. J Am Coll Cardiol.

[11] Kim SM, Kim YN, Choe YH (2013). Adenosine-stress dynamic myocardial perfusion imaging using 128-slice dual-source CT: optimization of the CT protocol to reduce the radiation dose. Int J Cardiovasc Imaging.

[12] Fujita M, Kitagawa K, Ito T, Shiraishi Y, Kurobe Y, Nagata M, Ishida M, Sakuma H (2014). Dose reduction in dynamic CT stress myocardial perfusion imaging: comparison of 80-kV/370-mAs and 100-kV/300-mAs protocols. Eur Radiol.

[13] Jakobs TF, Becker CR, Ohnesorge B, Flohr T, Suess C, Schoepf UJ, Reiser MF (2002). Multislice helical CT of the heart with retrospective ECG gating: reduction of radiation exposure by ECG-controlled tube current modulation. Eur Radiol.

[14] Greupner J, Zimmermann E, Grohmann A, Dubel HP, Althoff TF, Borges AC (2012). Head-to-head comparison of left ventricular function assessment with 64-row computed tomography, biplane left cineventriculography, and both 2- and 3-dimensional transthoracic echocardiography: comparison with magnetic resonance imaging as the reference standard. J Am Coll Cardiol.

[15] Raman SV, Shah M, McCarthy B, Garcia A, Ferketich AK (2006). Multi-detector row cardiac computed tomography accurately quantifies right and left ventricular size and function compared with cardiac magnetic resonance. Am Heart J.

[16] Blankstein R, Di Carli MF (2010). Integration of coronary anatomy and myocardial perfusion imaging. Nat Rev Cardiol.

[17] Boden WE, O’Rourke RA, Teo KK, Hartigan PM, Maron DJ, Kostuk WJ, Knudtson M, Dada M, Casperson P, Harris CL, Chaitman BR, Shaw L, Gosselin G, Nawaz S, Title LM, Gau G, Blaustein AS, Booth DC, Bates ER, Spertus JA, Berman DS, Mancini GB, Weintraub WS, COURAGE Trial Research Group (2007). Optimal medical therapy with or without PCI for stable coronary disease. N Engl J Med.

[18] Tonino PA, De Bruyne B, Pijls NH, Siebert U, Ikeno F, van’t Veer M (2009). Fractional flow reserve versus angiography for guiding percutaneous coronary intervention. N Engl J Med.

[19] De Bruyne B, Fearon WF, Pijls NH, Barbato E, Tonino P, Piroth Z (2014). Fractional flow reserve-guided PCI for stable coronary artery disease. N Engl J Med.

[20] Takx RA, Blomberg BA, El Aidi H, Habets J, de Jong PA, Nagel E et al. Diagnostic accuracy of stress myocardial perfusion imaging compared to invasive coronary angiography with fractional flow reserve meta-analysis. Circ Cardiovasc Imaging. 2015;8(1). 10.1161/circimaging.114.002666. 10.1161/CIRCIMAGING.114.002666 25596143

[21] Thompson RC, O’Keefe JH, McGhie AI, Bybee KA, Thompson EC, Bateman TM (2018). Reduction of SPECT MPI radiation dose using contemporary protocols and technology. JACC Cardiovasc Imaging.

[22] Carpeggiani C, Picano E, Brambilla M, Michelassi C, Knuuti J, Kauffman P (2017). Variability of radiation doses of cardiac diagnostic imaging tests: the RADIO-EVINCI study (RADIationdOse subproject of the EVINCI study). BMC Cardiovasc Disord.

[23] Huang JY, Huang CK, Yen RF, Wu HY, Tu YK, Cheng MF, Lu CC, Tzen KY, Chien KL, Wu YW (2016). Diagnostic performance of attenuation-corrected myocardial perfusion imaging for coronary artery disease: a systematic review and meta-analysis. Journal of Nuclear Medicine: official publication, Society of Nuclear Medicine..

[24] Worden NE, Lindower PD, Burns TL, Chatterjee K, Weiss RM (2015). A second look with prone SPECT myocardial perfusion imaging reduces the need for angiography in patients at low risk for cardiac death or MI. J Nucl Cardiol.

[25] Nakazato R, Berman DS, Hayes SW, Fish M, Padgett R, Xu Y, Lemley M, Baavour R, Roth N, Slomka PJ (2013). Myocardial perfusion imaging with a solid-state camera: simulation of a very low dose imaging protocol. Journal of Nuclear Medicine: official publication, Society of Nuclear Medicine..

[26] Wolk MJ, Bailey SR, Doherty JU, Douglas PS, Hendel RC, Kramer CM, Min JK, Patel MR, Rosenbaum L, Shaw LJ, Stainback RF, Allen JM, American College of Cardiology Foundation Appropriate Use Criteria Task Force (2014). ACCF/AHA/ASE/ASNC/HFSA/HRS/SCAI/SCCT/SCMR/STS 2013 multimodality appropriate use criteria for the detection and risk assessment of stable ischemic heart disease: a report of the American College of Cardiology Foundation Appropriate Use Criteria Task Force, American Heart Association, American Society of Echocardiography, American Society of Nuclear Cardiology, Heart Failure Society of America, Heart Rhythm Society, Society for Cardiovascular Angiography and Interventions, Society of Cardiovascular Computed Tomography, Society for Cardiovascular Magnetic Resonance, and Society of Thoracic Surgeons. J Am Coll Cardiol.

[27] Udelson JE, Coleman PS, Metherall J, Pandian NG, Gomez AR, Griffith JL, Shea NL, Oates E, Konstam MA (1994). Predicting recovery of severe regional ventricular dysfunction. Comparison of resting scintigraphy with 201Tl and 99mTc-sestamibi. Circulation.

[28] Agostini D, Roule V, Nganoa C, Roth N, Baavour R, Parienti JJ, et al. First validation of myocardial flow reserve assessed by dynamic (99m)Tc-sestamibi CZT-SPECT camera: head to head comparison with (15)O-water PET and fractional flow reserve in patients with suspected coronary artery disease. The WATERDAY study. Eur J Nucl Med Mol Imaging. 2018; 10.1007/s00259-018-3958-7. 10.1007/s00259-018-3958-7 PMC5953996 29497801

[29] Alessio AM, Bassingthwaighte JB, Glenny R, Caldwell JH (2013). Validation of an axially distributed model for quantification of myocardial blood flow using (1)(3)N-ammonia PET. J Nucl Cardiol.

[30] Gullberg GT, Shrestha UM, Seo Y. Dynamic cardiac PET imaging: technological improvements advancing future cardiac health. J Nucl Cardiol. 2018; 10.1007/s12350-018-1201-3. 10.1007/s12350-018-1201-3 PMC6068005 29388118

[31] Mc Ardle BA, Dowsley TF, de Kemp RA, Wells GA, Beanlands RS (2012). Does rubidium-82 PET have superior accuracy to SPECT perfusion imaging for the diagnosis of obstructive coronary disease?: a systematic review and meta-analysis. J Am Coll Cardiol.

[32] Hamon M, Fau G, Nee G, Ehtisham J, Morello R, Hamon M (2010). Meta-analysis of the diagnostic performance of stress perfusion cardiovascular magnetic resonance for detection of coronary artery disease. J Cardiovasc Magn Reson.

[33] Greenwood JP, Maredia N, Younger JF, Brown JM, Nixon J, Everett CC, Bijsterveld P, Ridgway JP, Radjenovic A, Dickinson CJ, Ball SG, Plein S (2012). Cardiovascular magnetic resonance and single-photon emission computed tomography for diagnosis of coronary heart disease (CE-MARC): a prospective trial. Lancet.

[34] Jaarsma C, Leiner T, Bekkers SC, Crijns HJ, Wildberger JE, Nagel E, Nelemans PJ, Schalla S (2012). Diagnostic performance of noninvasive myocardial perfusion imaging using single-photon emission computed tomography, cardiac magnetic resonance, and positron emission tomography imaging for the detection of obstructive coronary artery disease: a meta-analysis. J Am Coll Cardiol.

[35] Schwitter J, Wacker CM, van Rossum AC, Lombardi M, Al-Saadi N, Ahlstrom H (2008). MR-IMPACT: comparison of perfusion-cardiac magnetic resonance with single-photon emission computed tomography for the detection of coronary artery disease in a multicentre, multivendor, randomized trial. Eur Heart J.

[36] Greenwood JP, Motwani M, Maredia N, Brown JM, Everett CC, Nixon J, Bijsterveld P, Dickinson CJ, Ball SG, Plein S (2014). Comparison of cardiovascular magnetic resonance and single-photon emission computed tomography in women with suspected coronary artery disease from the Clinical Evaluation of Magnetic Resonance Imaging in Coronary Heart Disease (CE-MARC) trial. Circulation.

[37] Schwitter J, Wacker CM, Wilke N, Al-Saadi N, Sauer E, Huettle K (2013). MR-IMPACT II: magnetic resonance imaging for myocardial perfusion assessment in coronary artery disease trial: perfusion-cardiac magnetic resonance vs. single-photon emission computed tomography for the detection of coronary artery disease: a comparative multicentre, multivendor trial. Eur Heart J.

[38] Cury RC, Kitt TM, Feaheny K, Blankstein R, Ghoshhajra BB, Budoff MJ, Leipsic J, Min JK, Akin J, George RT (2015). A randomized, multicenter, multivendor study of myocardial perfusion imaging with regadenoson CT perfusion vs single photon emission CT. J Cardiovasc Comput Tomogr.

[39] Blankstein R, Shturman LD, Rogers IS, Rocha-Filho JA, Okada DR, Sarwar A, Soni AV, Bezerra H, Ghoshhajra BB, Petranovic M, Loureiro R, Feuchtner G, Gewirtz H, Hoffmann U, Mamuya WS, Brady TJ, Cury RC (2009). Adenosine-induced stress myocardial perfusion imaging using dual-source cardiac computed tomography. J Am Coll Cardiol.

[40] Rocha-Filho JA, Blankstein R, Shturman LD, Bezerra HG, Okada DR, Rogers IS, Ghoshhajra B, Hoffmann U, Feuchtner G, Mamuya WS, Brady TJ, Cury RC (2010). Incremental value of adenosine-induced stress myocardial perfusion imaging with dual-source CT at cardiac CT angiography. Radiology.

[41] Feuchtner G, Goetti R, Plass A, Wieser M, Scheffel H, Wyss C, Stolzmann P, Donati O, Schnabl J, Falk V, Alkadhi H, Leschka S, Cury RC (2011). Adenosine stress high-pitch 128-slice dual-source myocardial computed tomography perfusion for imaging of reversible myocardial ischemia: comparison with magnetic resonance imaging. Circ Cardiovasc Imaging.

[42] Cury RC, Magalhaes TA, Paladino AT, Shiozaki AA, Perini M, Senra T (2011). Dipyridamole stress and rest transmural myocardial perfusion ratio evaluation by 64 detector-row computed tomography. J Cardiovasc Comput Tomogr.

[43] Ko BS, Cameron JD, Meredith IT, Leung M, Antonis PR, Nasis A, Crossett M, Hope SA, Lehman SJ, Troupis J, DeFrance T, Seneviratne SK (2012). Computed tomography stress myocardial perfusion imaging in patients considered for revascularization: a comparison with fractional flow reserve. Eur Heart J.

[44] Ko SM, Choi JW, Hwang HK, Song MG, Shin JK, Chee HK (2012). Diagnostic performance of combined noninvasive anatomic and functional assessment with dual-source CT and adenosine-induced stress dual-energy CT for detection of significant coronary stenosis. AJR Am J Roentgenol.

[45] George RT, Arbab-Zadeh A, Miller JM, Vavere AL, Bengel FM, Lardo AC, Lima JAC (2012). Computed tomography myocardial perfusion imaging with 320-row detector computed tomography accurately detects myocardial ischemia in patients with obstructive coronary artery disease. Circ Cardiovasc Imaging.

[46] Nasis A, Ko BS, Leung MC, Antonis PR, Nandurkar D, Wong DT, Kyi L, Cameron JD, Troupis JM, Meredith IT, Seneviratne SK (2013). Diagnostic accuracy of combined coronary angiography and adenosine stress myocardial perfusion imaging using 320-detector computed tomography: pilot study. Eur Radiol.

[47] Rochitte CE, George RT, Chen MY, Arbab-Zadeh A, Dewey M, Miller JM, Niinuma H, Yoshioka K, Kitagawa K, Nakamori S, Laham R, Vavere AL, Cerci RJ, Mehra VC, Nomura C, Kofoed KF, Jinzaki M, Kuribayashi S, de Roos A, Laule M, Tan SY, Hoe J, Paul N, Rybicki FJ, Brinker JA, Arai AE, Cox C, Clouse ME, di Carli MF, Lima JAC (2014). Computed tomography angiography and perfusion to assess coronary artery stenosis causing perfusion defects by single photon emission computed tomography: the CORE320 study. Eur Heart J.

[48] Osawa K, Miyoshi T, Koyama Y, Hashimoto K, Sato S, Nakamura K, Nishii N, Kohno K, Morita H, Kanazawa S, Ito H (2014). Additional diagnostic value of first-pass myocardial perfusion imaging without stress when combined with 64-row detector coronary CT angiography in patients with coronary artery disease. Heart.

[49] Kido T, Kurata A, Higashino H, Inoue Y, Kanza RE, Okayama H, Higaki J, Murase K, Mochizuki T (2008). Quantification of regional myocardial blood flow using first-pass multidetector-row computed tomography and adenosine triphosphate in coronary artery disease. Circ J.

[50] Bastarrika G, Ramos-Duran L, Rosenblum MA, Kang DK, Rowe GW, Schoepf UJ (2010). Adenosine-stress dynamic myocardial CT perfusion imaging: initial clinical experience. Investig Radiol.

[51] Ho KT, Chua KC, Klotz E, Panknin C (2010). Stress and rest dynamic myocardial perfusion imaging by evaluation of complete time-attenuation curves with dual-source CT. JACC Cardiovasc Imaging.

[52] Bamberg F, Becker A, Schwarz F, Marcus RP, Greif M, von Ziegler F, Blankstein R, Hoffmann U, Sommer WH, Hoffmann VS, Johnson TRC, Becker HCR, Wintersperger BJ, Reiser MF, Nikolaou K (2011). Detection of hemodynamically significant coronary artery stenosis: incremental diagnostic value of dynamic CT-based myocardial perfusion imaging. Radiology.

[53] So A, Wisenberg G, Islam A, Amann J, Romano W, Brown J, Humen D, Jablonsky G, Li JY, Hsieh J, Lee TY (2012). Non-invasive assessment of functionally relevant coronary artery stenoses with quantitative CT perfusion: preliminary clinical experiences. Eur Radiol.

[54] Wang Y, Qin L, Shi X, Zeng Y, Jing H, Schoepf UJ, Jin Z (2012). Adenosine-stress dynamic myocardial perfusion imaging with second-generation dual-source CT: comparison with conventional catheter coronary angiography and SPECT nuclear myocardial perfusion imaging. AJR Am J Roentgenol.

[55] Weininger M, Schoepf UJ, Ramachandra A, Fink C, Rowe GW, Costello P, Henzler T (2012). Adenosine-stress dynamic real-time myocardial perfusion CT and adenosine-stress first-pass dual-energy myocardial perfusion CT for the assessment of acute chest pain: initial results. Eur J Radiol.

[56] Rossi A, Uitterdijk A, Dijkshoorn M, Klotz E, Dharampal A, van Straten M, van der Giessen WJ, Mollet N, van Geuns RJ, Krestin GP, Duncker DJ, de Feyter PJ, Merkus D (2013). Quantification of myocardial blood flow by adenosine-stress CT perfusion imaging in pigs during various degrees of stenosis correlates well with coronary artery blood flow and fractional flow reserve. Eur Heart J Cardiovasc Imaging.

[57] Greif M, von Ziegler F, Bamberg F, Tittus J, Schwarz F, D’Anastasi M, Marcus RP, Schenzle J, Becker C, Nikolaou K, Becker A (2013). CT stress perfusion imaging for detection of haemodynamically relevant coronary stenosis as defined by FFR. Heart.

[58] Huber AM, Leber V, Gramer BM, Muenzel D, Leber A, Rieber J, Schmidt M, Vembar M, Hoffmann E, Rummeny E (2013). Myocardium: dynamic versus single-shot CT perfusion imaging. Radiology.

[59] Bamberg F, Marcus RP, Becker A, Hildebrandt K, Bauner K, Schwarz F, Greif M, von Ziegler F, Bischoff B, Becker HC, Johnson TR, Reiser MF, Nikolaou K, Theisen D (2014). Dynamic myocardial CT perfusion imaging for evaluation of myocardial ischemia as determined by MR imaging. JACC Cardiovasc Imaging.

[60] Magalhaes TA, Kishi S, George RT, Arbab-Zadeh A, Vavere AL, Cox C (2015). Combined coronary angiography and myocardial perfusion by computed tomography in the identification of flow-limiting stenosis—the CORE320 study: an integrated analysis of CT coronary angiography and myocardial perfusion. J Cardiovasc Comput Tomogr.

[61] Baxa J, Hromadka M, Sedivy J, Stepankova L, Molacek J, Schmidt B (2015). Regadenoson-stress dynamic myocardial perfusion improves diagnostic performance of CT angiography in assessment of intermediate coronary artery stenosis in asymptomatic patients. Biomed Res Int.

[62] Wichmann JL, Meinel FG, Schoepf UJ, Varga-Szemes A, Muscogiuri G, Cannao PM (2016). Semiautomated global quantification of left ventricular myocardial perfusion at stress dynamic CT: diagnostic accuracy for detection of territorial myocardial perfusion deficits compared to visual assessment. Acad Radiol.

[63] Kachenoura N, Gaspar T, Lodato JA, Bardo DM, Newby B, Gips S (2009). Combined assessment of coronary anatomy and myocardial perfusion using multidetector computed tomography for the evaluation of coronary artery disease. Am J Cardiol.

[64] George RT, Arbab-Zadeh A, Miller JM, Kitagawa K, Chang HJ, Bluemke DA, Becker L, Yousuf O, Texter J, Lardo AC, Lima JAC (2009). Adenosine stress 64- and 256-row detector computed tomography angiography and perfusion imaging: a pilot study evaluating the transmural extent of perfusion abnormalities to predict atherosclerosis causing myocardial ischemia. Circ Cardiovasc Imaging.

[65] Tanabe Y, Kido T, Uetani T, Kurata A, Kono T, Ogimoto A, Miyagawa M, Soma T, Murase K, Iwaki H, Mochizuki T (2016). Differentiation of myocardial ischemia and infarction assessed by dynamic computed tomography perfusion imaging and comparison with cardiac magnetic resonance and single-photon emission computed tomography. Eur Radiol.

[66] Cury RC, Magalhaes TA, Borges AC, Shiozaki AA, Lemos PA, Junior JS (2010). Dipyridamole stress and rest myocardial perfusion by 64-detector row computed tomography in patients with suspected coronary artery disease. Am J Cardiol.

[67] Mahnken AH, Lautenschlager S, Fritz D, Koos R, Scheuering M (2008). Perfusion weighted color maps for enhanced visualization of myocardial infarction by MSCT: preliminary experience. Int J Cardiovasc Imaging.

[68] Carrascosa P, Capunay C (2017). Myocardial CT perfusion imaging for ischemia detection. Cardiovasc Diagn Ther.

[69] Thomas DM, Larson CW, Cheezum MK, Villines TC, Branch KR, Blankstein R, Cury RC, Slim AM (2015). Rest-only myocardial CT perfusion in acute chest pain. South Med J.

[70] Zoghbi GJ, Dorfman TA, Iskandrian AE (2008). The effects of medications on myocardial perfusion. J Am Coll Cardiol.

[71] Hsiao EM, Rybicki FJ, Steigner M (2010). CT coronary angiography: 256-slice and 320-detector row scanners. Curr Cardiol Rep.

[72] Ebersberger U, Marcus RP, Schoepf UJ, Lo GG, Wang Y, Blanke P, Geyer LL, Gray JC, McQuiston AD, Cho YJ, Scheuering M, Canstein C, Nikolaou K, Hoffmann E, Bamberg F (2014). Dynamic CT myocardial perfusion imaging: performance of 3D semi-automated evaluation software. Eur Radiol.

[73] Bastarrika G, Ramos-Duran L, Schoepf UJ, Rosenblum MA, Abro JA, Brothers RL, Zubieta JL, Chiaramida SA, Kang DK (2010). Adenosine-stress dynamic myocardial volume perfusion imaging with second generation dual-source computed tomography: concepts and first experiences. J Cardiovasc Comput Tomogr.

[74] Ruzsics B, Schwarz F, Schoepf UJ, Lee YS, Bastarrika G, Chiaramida SA, Costello P, Zwerner PL (2009). Comparison of dual-energy computed tomography of the heart with single photon emission computed tomography for assessment of coronary artery stenosis and of the myocardial blood supply. Am J Cardiol.

[75] Ruzsics B, Lee H, Powers ER, Flohr TG, Costello P, Schoepf UJ (2008). Images in cardiovascular medicine. Myocardial ischemia diagnosed by dual-energy computed tomography: correlation with single-photon emission computed tomography. Circulation.

[76] Koonce JD, Vliegenthart R, Schoepf UJ, Schmidt B, Wahlquist AE, Nietert PJ, Bastarrika G, Flohr TG, Meinel FG (2014). Accuracy of dual-energy computed tomography for the measurement of iodine concentration using cardiac CT protocols: validation in a phantom model. Eur Radiol.

[77] Danad I, Fayad ZA, Willemink MJ, Min JK (2015). New applications of cardiac computed tomography: dual-energy, spectral, and molecular CT imaging. JACC Cardiovasc Imaging.

[78] Scheske JA, O’Brien JM, Earls JP, Min JK, LaBounty TM, Cury RC (2013). Coronary artery imaging with single-source rapid kilovolt peak-switching dual-energy CT. Radiology.

[79] Yu L, Christner JA, Leng S, Wang J, Fletcher JG, McCollough CH (2011). Virtual monochromatic imaging in dual-source dual-energy CT: radiation dose and image quality. Med Phys.

[80] So A, Hsieh J, Narayanan S, Thibault JB, Imai Y, Dutta S, Leipsic J, Min J, LaBounty T, Lee TY (2012). Dual-energy CT and its potential use for quantitative myocardial CT perfusion. J Cardiovasc Comput Tomogr.

[81] Kang DK, Schoepf UJ, Bastarrika G, Nance JW, Abro JA, Ruzsics B (2010). Dual-energy computed tomography for integrative imaging of coronary artery disease: principles and clinical applications. Semin Ultrasound CT MR.

[82] Wang R, Yu W, Wang Y, He Y, Yang L, Bi T, Jiao J, Wang Q, Chi L, Yu Y, Zhang Z (2011). Incremental value of dual-energy CT to coronary CT angiography for the detection of significant coronary stenosis: comparison with quantitative coronary angiography and single photon emission computed tomography. Int J Cardiovasc Imaging.

[83] Ko SM, Choi JW, Song MG, Shin JK, Chee HK, Chung HW, Kim DH (2011). Myocardial perfusion imaging using adenosine-induced stress dual-energy computed tomography of the heart: comparison with cardiac magnetic resonance imaging and conventional coronary angiography. Eur Radiol.

[84] Kim SM, Chang SA, Shin W, Choe YH (2014). Dual-energy CT perfusion during pharmacologic stress for the assessment of myocardial perfusion defects using a second-generation dual-source CT: a comparison with cardiac magnetic resonance imaging. J Comput Assist Tomogr.

[85] Ko SM, Park JH, Hwang HK, Song MG (2014). Direct comparison of stress- and rest-dual-energy computed tomography for detection of myocardial perfusion defect. Int J Cardiovasc Imaging.

[86] Albrecht MH, Trommer J, Wichmann JL, Scholtz JE, Martin SS, Lehnert T, Vogl TJ, Bodelle B (2016). Comprehensive comparison of virtual monoenergetic and linearly blended reconstruction techniques in third-generation dual-source dual-energy computed tomography angiography of the thorax and abdomen. Investig Radiol.

[87] Rodriguez-Granillo GA, Carrascosa P, Cipriano S, de Zan M, Deviggiano A, Capunay C, Cury RC (2015). Myocardial signal density levels and beam-hardening artifact attenuation using dual-energy computed tomography. Clin Imaging.

[88] Meinel FG, De Cecco CN, Schoepf UJ, Nance JW, Silverman JR, Flowers BA (2014). First-arterial-pass dual-energy CT for assessment of myocardial blood supply: do we need rest, stress, and delayed acquisition? Comparison with SPECT. Radiology.

[89] Bettencourt N, Ferreira ND, Leite D, Carvalho M, Ferreira WDS, Schuster A (2013). CAD detection in patients with intermediate-high pre-test probability: low-dose CT delayed enhancement detects ischemic myocardial scar with moderate accuracy but does not improve performance of a stress-rest CT perfusion protocol. JACC Cardiovasc Imaging.

[90] Carrascosa P, Capunay C, Rodriguez-Granillo GA, Deviggiano A, Vallejos J, Leipsic JA (2014). Substantial iodine volume load reduction in CT angiography with dual-energy imaging: insights from a pilot randomized study. Int J Cardiovasc Imaging.

[91] Carrascosa P, Leipsic JA, Capunay C, Deviggiano A, Vallejos J, Goldsmit A, Rodriguez-Granillo GA (2015). Monochromatic image reconstruction by dual energy imaging allows half iodine load computed tomography coronary angiography. Eur J Radiol.

[92] Secchi F, De Cecco CN, Spearman JV, Silverman JR, Ebersberger U, Sardanelli F (2015). Monoenergetic extrapolation of cardiac dual energy CT for artifact reduction. Acta Radiol (Stockholm, Sweden : 1987).

[93] Yamada M, Jinzaki M, Kuribayashi S, Imanishi N, Funato K, Aiso S (2012). Beam-hardening correction for virtual monochromatic imaging of myocardial perfusion via fast-switching dual-kVp 64-slice computed tomography: a pilot study using a human heart specimen. Circ J.

[94] So A, Lee TY, Imai Y, Narayanan S, Hsieh J, Kramer J, Procknow K, Leipsic J, LaBounty T, Min J (2011). Quantitative myocardial perfusion imaging using rapid kVp switch dual-energy CT: preliminary experience. J Cardiovasc Comput Tomogr.

[95] Rogers IS, Cury RC, Blankstein R, Shapiro MD, Nieman K, Hoffmann U, Brady TJ, Abbara S (2010). Comparison of postprocessing techniques for the detection of perfusion defects by cardiac computed tomography in patients presenting with acute ST-segment elevation myocardial infarction. J Cardiovasc Comput Tomogr.

[96] Stanton CL, Haramati LB, Berko NS, Travin MI, Jain VR, Jacobi AH, Burton WB, Levsky JM (2011). Normal myocardial perfusion on 64-detector resting cardiac CT. J Cardiovasc Comput Tomogr.

[97] Nieman K, Cury RC, Ferencik M, Nomura CH, Abbara S, Hoffmann U, Gold HK, Jang IK, Brady TJ (2006). Differentiation of recent and chronic myocardial infarction by cardiac computed tomography. Am J Cardiol.

[98] Mahmarian JJ, Fenimore NL, Marks GF, Francis MJ, Morales-Ballejo H, Verani MS, Pratt CM (1994). Transdermal nitroglycerin patch therapy reduces the extent of exercise-induced myocardial ischemia: results of a double-blind, placebo-controlled trial using quantitative thallium-201 tomography. J Am Coll Cardiol.

[99] Reyes E, Stirrup J, Roughton M, D’Souza S, Underwood SR, Anagnostopoulos C (2010). Attenuation of adenosine-induced myocardial perfusion heterogeneity by atenolol and other cardioselective beta-adrenoceptor blockers: a crossover myocardial perfusion imaging study. J Nucl Med.

[100] Saeed M, Bremerich J, Wendland MF, Wyttenbach R, Weinmann HJ, Higgins CB (1999). Reperfused myocardial infarction as seen with use of necrosis-specific versus standard extracellular MR contrast media in rats. Radiology.

[101] Wang J, Xiang B, Lin HY, Liu H, Freed D, Arora RC, Tian G (2015). Differential MR delayed enhancement patterns of chronic myocardial infarction between extracellular and intravascular contrast media. PLoS One.

[102] Wang R, Zhang Z, Xu L, Ma Q, He Y, Lu D, Yu W, Fan Z (2011). Low dose prospective ECG-gated delayed enhanced dual-source computed tomography in reperfused acute myocardial infarction comparison with cardiac magnetic resonance. Eur J Radiol.

[103] Jacquier A, Boussel L, Amabile N, Bartoli JM, Douek P, Moulin G, Paganelli F, Saeed M, Revel D, Croisille P (2008). Multidetector computed tomography in reperfused acute myocardial infarction. Assessment of infarct size and no-reflow in comparison with cardiac magnetic resonance imaging. Investig Radiol.

[104] Sato A, Nozato T, Hikita H, Akiyama D, Nishina H, Hoshi T, Aihara H, Kakefuda Y, Watabe H, Hiroe M, Aonuma K (2012). Prognostic value of myocardial contrast delayed enhancement with 64-slice multidetector computed tomography after acute myocardial infarction. J Am Coll Cardiol.

[105] Andreini D, Pontone G, Pepi M, Ballerini G, Bartorelli AL, Magini A, Quaglia C, Nobili E, Agostoni P (2007). Diagnostic accuracy of multidetector computed tomography coronary angiography in patients with dilated cardiomyopathy. J Am Coll Cardiol.

[106] Guo YK, Gao HL, Zhang XC, Wang QL, Yang ZG, Ma ES (2010). Accuracy and reproducibility of assessing right ventricular function with 64-section multi-detector row CT: comparison with magnetic resonance imaging. Int J Cardiol.

[107] Halliburton SS, Abbara S, Chen MY, Gentry R, Mahesh M, Raff GL, Shaw LJ, Hausleiter J, Society of Cardiovascular Computed Tomography (2011). SCCT guidelines on radiation dose and dose-optimization strategies in cardiovascular CT. J Cardiovasc Comput Tomogr.

[108] Lu JG, Lv B, Chen XB, Tang X, Jiang SL, Dai RP (2010). What is the best contrast injection protocol for 64-row multi-detector cardiac computed tomography?. Eur J Radiol.

[109] Skali H, Schulman AR, Dorbala S. 18F-FDG PET/CT for the assessment of myocardial sarcoidosis. Curr Cardiol Rep. 2013;15(4). 10.1007/s11886-013-0370-6. PMC4009625 23544184

[110] Bokhari S, Shahzad R, Castano A, Maurer MS (2014). Nuclear imaging modalities for cardiac amyloidosis. J Nucl Cardiol.

[111] Lee HJ, Im DJ, Youn JC, Chang S, Suh YJ, Hong YJ, Kim YJ, Hur J, Choi BW (2016). Myocardial extracellular volume fraction with dual-energy equilibrium contrast-enhanced cardiac CT in nonischemic cardiomyopathy: a prospective comparison with cardiac MR imaging. Radiology.

[112] Kellman P, Wilson JR, Xue H, Ugander M, Arai AE (2012). Extracellular volume fraction mapping in the myocardium, part 1: evaluation of an automated method. J Cardiovasc Magnetic Resonance: official journal of the Society for Cardiovascular Magn Reson.

[113] Nacif MS, Kawel N, Lee JJ, Chen X, Yao J, Zavodni A, Sibley CT, Lima JAC, Liu S, Bluemke DA (2012). Interstitial myocardial fibrosis assessed as extracellular volume fraction with low-radiation-dose cardiac CT. Radiology.

[114] Buss SJ, Schulz F, Mereles D, Hosch W, Galuschky C, Schummers G, Stapf D, Hofmann N, Giannitsis E, Hardt SE, Kauczor HU, Katus HA, Korosoglou G (2014). Quantitative analysis of left ventricular strain using cardiac computed tomography. Eur J Radiol.

